# Modulating the Precursor and Terpene Synthase Supply for the Whole-Cell Biocatalytic Production of the Sesquiterpene (+)-Zizaene in a Pathway Engineered *E. coli*

**DOI:** 10.3390/genes10060478

**Published:** 2019-06-24

**Authors:** Francisco Aguilar, Thomas Scheper, Sascha Beutel

**Affiliations:** Institute of Technical Chemistry, Leibniz University of Hannover, Callinstr. 5, 30167 Hannover, Germany; aguilar@iftc.uni-hannover.de (F.A.); scheper@iftc.uni-hannover.de (T.S.)

**Keywords:** khusimene, (+)-zizaene, vetiver essential oil, khusimol, sesquiterpenes, microbial production, metabolic engineering

## Abstract

The vetiver essential oil from *Chrysopogon zizanioides* contains fragrant sesquiterpenes used widely in the formulation of nearly 20% of men’s cosmetics. The growing demand and issues in the supply have raised interest in the microbial production of the sesquiterpene khusimol, the main compound of the vetiver essential oil due to its woody smell. In this study, we engineered the biosynthetic pathway for the production of (+)-zizaene, the immediate precursor of khusimol. A systematic approach of metabolic engineering in *Escherichia coli* was applied to modulate the critical bottlenecks of the metabolic flux towards (+)-zizaene. Initially, production of (+)-zizaene was possible with the endogenous methylerythritol phosphate pathway and the codon-optimized zizaene synthase (ZS). Raising the precursor E,E-farnesyl diphosphate supply through the mevalonate pathway improved the (+)-zizaene titers 2.7-fold, although a limitation of the ZS supply was observed. To increase the ZS supply, distinct promoters were tested for the expression of the *ZS* gene, which augmented 7.2-fold in the (+)-zizaene titers. Final metabolic enhancement for the ZS supply by using a multi-plasmid strain harboring multiple copies of the *ZS* gene improved the (+)-zizaene titers 1.3-fold. The optimization of the fermentation conditions increased the (+)-zizaene titers 2.2-fold, achieving the highest (+)-zizaene titer of 25.09 mg L^−1^. This study provides an alternative strategy to enhance the terpene synthase supply for the engineering of isoprenoids. Moreover, it demonstrates the development of a novel microbial platform for the sustainable production of fragrant molecules for the cosmetic industry.

## 1. Introduction

The cosmetic industry has extensively used essential oils for the formulation of perfumes due to their complex mixture of fragrant hydrocarbons such as ketones, ethers, esters, alcohols, aldehydes, and terpenoids [1]. From these natural products, terpenes such as volatile monoterpenes, sesquiterpenes, and diterpenes are the main constituents, which confer the specific odor to each essential oil [2].

Among the distinct essential oils, the vetiver essential oil (VEO) from the *Ch. zizanioides* grass has drawn growing attention, because it is used in nearly 20% of men’s luxury perfumes [3]. Characterized by its dark woody smell, VEO holds a world production of 300–350 tons per year [4] with prices ranging from $380–$500 per kg in 2018 [5] and expected growth in the market of $169.5 million for 2022 [6]. Industrial production of VEO is performed traditionally by steam distillation from vetiver roots, harvested after 12–18 months of growth and at 1–2 m length; usually, this process attains an extraction yield of 0.2–3% and can be improved to 4% through supercritical fluid extraction [3,7]. The recurrent shortages in the supply of VEO from the main producer, Haiti due to rainfalls and earthquakes [3,5], and the increasing market demand has raised the interest towards a reliable biotechnological production of the main constituent of VEO, the sesquiterpene khusimol, which grants the distinctive woody smell.

Similar to most plant sesquiterpenes, the biosynthesis of khusimol initiates from the glycolysis cycle, which generates the precursors’ glyceraldehyde 3-phosphate (GAP) and pyruvate for the plastidial methylerythritol phosphate (MEP) pathway, and acetyl coenzyme A (Acetyl-CoA) for the cytoplasmic mevalonate (MEV) pathway [8]. Both pathways converge and catalyze the formation of the terpene precursors isopentenyl pyrophosphate (IPP) and isomer dimethylallyl pyrophosphate (DMAPP), which are further assembled by the farnesyl diphosphate synthase (*IspA*) to form the universal sesquiterpene substrate E,E-farnesyl diphosphate (FDP) [9]. In the (+)-zizaene (syn. khusimene) pathway, FDP is cyclized by the zizaene synthase (ZS) to yield (+)-zizaene and further hydroxylated to khusimol by a specific P450 cytochrome monooxygenase [10]. 

The biosynthetic pathway of khusimol should be possible in *E. coli* because of the positive results for the in vivo production of many sesquiterpenes [11]. Contrary to plants, *E. coli* produces endogenous FDP exclusively by the MEP pathway and is vital for the biosynthesis of the intermediates undecaprenyl diphosphate (UDP) and octaprenyl diphosphate (ODP) [12]. Accordingly, UDP is the direct precursor of peptidoglycans, used for cell wall formation [13], and ODP is involved in the respiratory chain for the biosynthesis of distinct quinones [14]. Consequently, amounts of FDP in *E. coli* are limited; thus, efforts have been made to engineer the MEP pathway to enhance the supply of FDP. As an example, the overexpression of the MEP pathway genes 1-Deoxy-D-xylulose 5-phosphate synthase (DXS) and 1-deoxy-D-xylulose 5-phosphate reductoisomerase (DXR) increased the FDP levels and improved the amounts of lycopene up to 22 mg L^−1^ [15]. Similarly, overexpression of the DXS synthase and additionally, the isoprenyl diphosphate isomerase (Idi) with the farnesyl pyrophosphate synthase (FPS) reached valerenadiene titers of 2.09 mg L^−1^ [12]. Although overexpression of the MEP pathway improved the precursor supply, the terpene titers did not reach industrial levels, possibly due to control mechanisms of *E. coli* or the formation of intermediates that limited the precursor supply [16,17].

As an alternative to raise the FDP supply, the heterologous MEV pathway was engineered in *E. coli* by Keasling and co-workers for the in vivo production of amorpha-4,11-diene [17]. The heterologous pathway involved the cloning of eight genes from *E. coli* and *Saccharomyces cerevisiae* into two operons: MevT operon converted acetyl-CoA to mevalonate and MBIS operon catalyzed mevalonate to IPP and DMAPP that led to FDP, yielding 112.2 mg L^−1^ as the highest amorpha-4,11-diene titer. The microbial platform was further enhanced for the production of bisabolene by optimizing the codon usage to *E. coli* for the *S. cerevisiae* genes and assembling the eight genes into one single operon under the control of the *lac*UV5 (MevT genes) and *trc* promoter (MBIS genes) [18]. As a result, the upgraded platform boosted the FDP supply and achieved titers of 900 mg L^−1^ of bisabolene.

The previously mentioned studies augmented the supply of the substrate FDP, which is further catalyzed in the terpene pathway by a terpene synthase (TPS), performing complex reactions that yield sesquiterpenes with precise structure and stereochemistry [19]. In the biosynthesis of khusimol, the TPS is the ZS that catalyzes the cyclization of FDP to the tricyclic (+)-zizaene. Its enzymatic characterization has been described by a recombinant codon-optimized variant fused to the small ubiquitin-related modifier (SUMO), which increased the solubility of ZS and expressed heterologously in *E. coli* [10]. Moreover, the catalytical specificity and enzymatic kinetics of the ZS enzyme have been further characterized [20].

In this study, we report the metabolic engineering of (+)-zizaene biosynthesis in *E. coli* strains. The engineering of the biosynthetic pathway involved the overexpression of the MEV pathway to enhance the FDP supply, the evaluation of promoters to efficiently express the ZS, and the engineering of multi-plasmid strains with multiple copies of ZS to boost the ZS supply. Moreover, optimization of the fermentation conditions and evaluation of *E. coli* strains were analyzed to improve the production of (+)-zizaene.

## 2. Materials and Methods

### 2.1. Engineering of Vectors and Strains

The description of vectors and strains is summarized in Table 1. The pETSUMO::ZIZ(co) vector was obtained from our previous study [10], and the sequence of the codon-optimized SUMO-fused *ZS* gene was described in GenBank accession KP231534. The vector pBbA5c-MevT(CO)-MBIS (CO, ispA), encoding the MEV pathway, was a gift from Jay Keasling & Taek Soon Lee (Addgene plasmid #35151). The pJbei-6411 vector for the expression with the arabinose operon was a gift from Taek Soon Lee (Addgene plasmid #47050).

The cloning procedures were carried out by the NEBuilder HiFi DNA Assembly method, and PCR reactions were performed with the Q5 HiFi DNA polymerase, both following the provider protocols (New England BioLabs, USA). Synthesis of oligonucleotides and sanger sequencing for clone confirmation were performed by Microsynth Seqlab (Germany), and primer sequences are described in Table 2.

For the production of (+)-zizaene by a single polycistronic vector, the *ZS* gene was cloned into the pMev vector, directly after the *IspA* gene to yield the pMevZS vector. In brief, the *ZS* gene was amplified from the pETZS vector by respective primers with complementary overhangs to the BamHI site of the pMev vector. Additionally, an internal ribosome entry site (IRES) (GATCTAGGAGGTA) from the MEV operon was cloned upstream from the *ZS* gene. Thereafter, the pMev vector was digested with BamHI, assembled with the *ZS* gene and *E. coli* NEB 10-beta competent cloning strain (New England BioLabs), which is specific for large vectors, was transformed with the pMev vector. For the testing of the P_BAD_ promoter through the pJbeiZS plasmid, the cytochrome P450 operon from pJbei-6411 vector was removed, and the *ZS* gene from the pETZS vector was inserted instead. For that, both the insert and backbone were amplified with complementary overhangs by PCR methods, seamlessly assembled, and *E. coli* TOP10 competent cloning strains (IBA, Germany) were transformed for respective screening. 

For the production of (+)-zizaene, *E. coli* expression strains were transformed or co-transformed with the respective plasmids by common methods [21]. An exception was the BZS+Mev strain, where the *E. coli* TOP10 competent cloning strain was used because expression with the arabinose operon requires a non-metabolizing arabinose strain [22].

### 2.2. Evaluation of the Engineered Strains for the Production (+)-zizaene

Cultivations of *E. coli* were performed by inoculating 100 µL of respective strains from glycerol stocks to 10 mL Lysogeny Broth (LB) pre-culture broths at 37 °C with respective antibiotics (kanamycin at 30 µg L^−1^ and, or chloramphenicol at 34 µg L^−1^) until the stationary phase. Furthermore, production cultures in sealed baffled shake-flasks with 35 mL of defined non-inducing broth (DNB) [24] were inoculated with 2% (v/v) of seed culture and grown at 37 °C on a rotatory shaker (150 rpm). After cultures reached a cell density of OD_600_ 0.6, (+)-zizaene production was induced by adding varying levels (0.1, 0.5, and 1.0 mM) of isopropyl-β-D-1-thiogalactopyranoside (IPTG) and an overlay of 4 mL iso-octane at 20 °C, (150 rpm) as a two-phase partitioning culture. After 24 h of induction, samples were taken promptly for analytical measurements that included dried cell weight (DCW), cell density, soluble ZS protein, and (+)-zizaene concentrations.

### 2.3. Optimization of the Fermentation Conditions for the Production (+)-zizaene

Cultivation and analytical procedures for media evaluation, optimization of growth temperature and media pH, and testing of *E. coli* strains were carried out as described before with the best performing strain. The evaluation of media comprised the defined media: defined non-inducing broth (DNB), M9 minimal medium (M9) [25], and a modified Aparicio defined medium (ADM) with the following composition (g L^−1^): (NH_4_)_2_SO_4_ 10.0, NaCl 1.2, K_2_SO_4_ 1.1, MgSO_4_·7H_2_O 0.15, K_2_HPO_4_ 9.3, KH_2_PO_4_ 2.03, CaCl_2_ 0.01, FeSO_4_·7H_2_O 0.001, and CuSO_4_·5H_2_O 0.001 [26]. Defined media were set to pH 7.2 and 5 g L^−1^ of glucose respectively. Additionally, the complex terrific broth (TB) [21] was used as a control. The factorial design for the pH and temperature optimization consisted of two factors with the following levels: pH (6.5, 7, and 7.5) and temperature (16, 20, 24, and 28 °C), for a total of 12 combinations with three replicates. Data were analyzed and plotted using the Minitab 16 software (USA).

### 2.4. Analytical Measurements

#### 2.4.1. ZS Protein Gel Electrophoresis Analysis

Production culture samples were normalized to an OD_600_ 2, centrifuged for 10 min (10,000× *g* at 4 °C) and free-media pellet was resuspended in 300 µL extraction buffer (50 mM MOPS, 150 mM NaCl, 5 mM DTT, 10% (v/v) glycerol, pH 7.5). Cells were disrupted through an ultrasonicator (Sartorius Labsonic, Goettingen, Germany) and soluble and insoluble protein fractions were separated after 30 min centrifugation (10,000× *g* at 4 °C). Protein fraction samples were mixed with 2X loading buffer, heated at 95 °C for 5 min and analyzed through a 10% sodium dodecyl sulfate polyacrylamide gel electrophoresis (SDS-PAGE).

#### 2.4.2. Identification and Measurement of (+)-zizaene by Gas Chromatography Analysis

Samples of 150 µL from the iso-octane overlay from production cultures were transferred to GC-vials for gas chromatography analysis and product identification was carried out through GC-MS using an Agilent 7890B system (Agilent, Santa Clara, CA, USA) with the following settings: sample injections of 0.5 µL on-column mode through a 30 m VF-WAXms capillary column (0.25 mm internal diameter I.D. × 0.25 μm, Agilent, USA); oven program of 40 °C for 3 min, increasing to 230 °C (10 °C min^−1^) and final hold of 10 min; helium 5.0 carrier gas at a constant gas flow of 1 mL min^−1^; temperature of 230 °C for ion source, and 150 °C for quadrupole; mass scan range of 33–300 *m*/*z* and ionization energy to 70 eV. Terpene products were identified by comparison of retention indices [27] and mass spectra of samples with authentic standards from the VEO and references from the mass spectral NIST 14 database.

Quantification of (+)-zizaene was performed by GC-FID with a GC-2010 plus GC system coupled to a flame ionization detector (Shimadzu, Japan) equipped with a 30 m Zebron ZB-Wax Plus column (0.25 mm I.D. × 0.25 μm, Phenomenex, USA). Samples of 1 µL were injected under a splitless mode with an injector temperature of 240 °C. The initial oven temperature was set to 40 °C for 20 s, ramped at 10 °C min^−1^ to 200 °C, held for 0.5 min, increased at a rate of 30 °C min^−1^ to 230 °C, and held finally for 2 min. Because the (+)-zizaene standard is not commercially available, an α-cedrene standard was used as an equivalent standard due to the relative abundance of the main peaks from the mass spectra as described in our previous report [20].

## 3. Results

### 3.1. Engineering the Production of (+)-zizaene by the MEP Pathway

**Figure 1:**
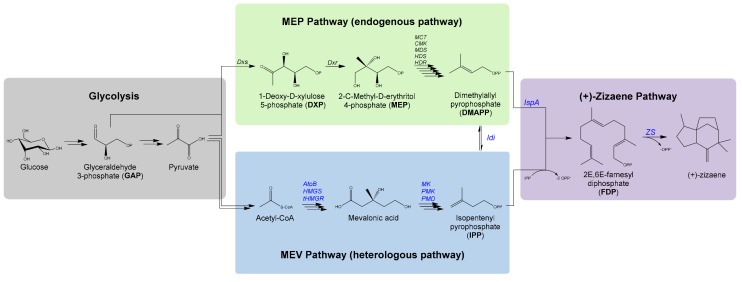
Biosynthetic pathway engineered for the production of (+)-zizaene in *E. coli* by the expression of the native methylerythritol phosphate (MEP) pathway, the heterologous MEV pathway and the (+)-zizaene pathway. Heterologous expressed enzymes highlighted in blue. The MEP pathway comprise the following enzymes: 1-Deoxy-D-xylulose 5-phosphate synthase (*Dxs*), 1-deoxy-D-xylulose 5-phosphate reductoisomerase (*Dxr*), MEP cytidyl transferase (*MCT*), cytidyl MEP kinase (*CMK*), MEP-2,4-cyclodiphosphate synthase (*MDS*), (E)-4-hydroxy-3-methylbut-2-enyl diphosphate synthase (*HDS*), and (E)-4-hydroxy-3-methylbut-2-enyl diphosphate reductase (*HDR*). The MEV heterologous pathway consisted of the following enzymes: acetyl-CoA acetyltransferase (*AtoB*), HMG-CoA synthase (HMGS), truncated HMG-CoA reductase (*tHMGR*), mevalonate kinase (MK), phosphomevalonate kinase (PMK), mevalonate diphosphate decarboxylase (PMD), and isoprenyl diphosphate isomerase (*Idi*). The (+)-zizaene pathway comprises the farnesyl diphosphate synthase (*IspA*) and the zizaene synthase (*ZS*).

In this study, we engineered the biosynthetic pathway in *E. coli* for the production of (+)-zizaene, using glucose as the sole carbon source (Figure 1). The key enzyme of the biosynthesis of (+)-zizaene is the ZS, which catalyzes the cyclization of the substrate FDP to (+)-zizaene. Due to the plant origin of TPS, most are expressed poorly in *E. coli*, leading to the formation of insoluble proteins (inclusion bodies) [28,29]. To improve TPS solubility, a recombinant ZS variant was used, which was codon-optimized for *E. coli* and fused with the SUMO moiety from our previous work [30].

**Figure 2:**
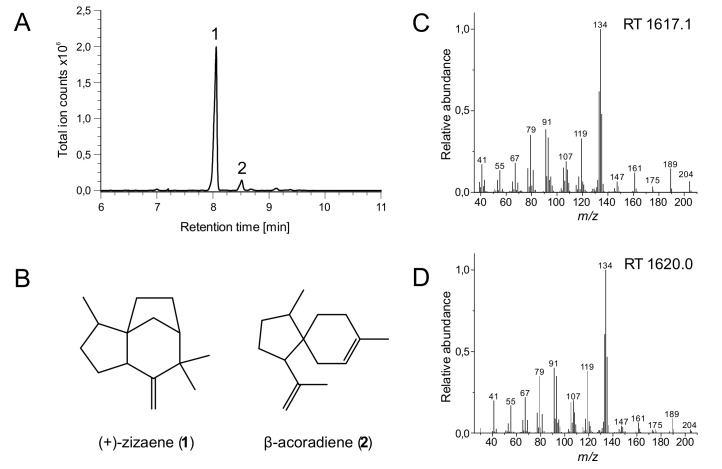
Identification of terpene products by GC–MS. (A) Total ion chromatogram. (B) Chemical structures of the identified products. (C) Mass spectra of (+)-zizaene from the sample of the ZS+MevZS strain. (D) Mass spectra of (+)-zizaene authentic standard from the vetiver essential oil. Comparison of mass spectra for β-acoradiene is shown in the Supplementary Table S1.

**Figure 3:**
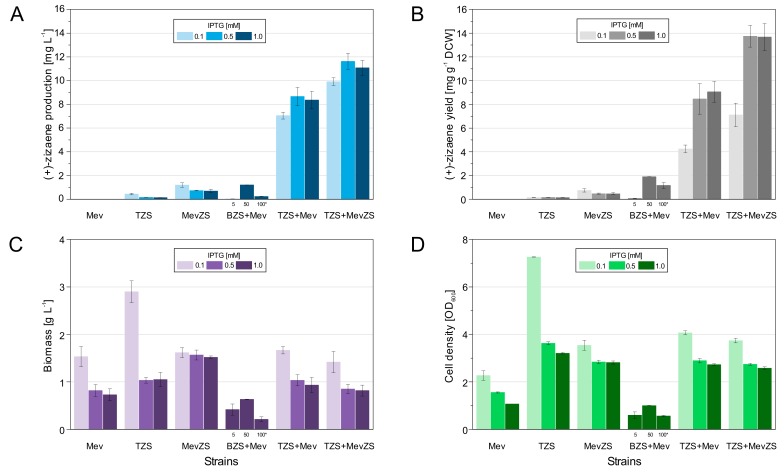
Comparison of the performance of the engineered *E. coli* BL21(DE3) strains induced at different IPTG levels. (**A**) (+)-zizaene titers. (**B**) (+)-zizaene yields. (**C**) biomass from cell dry weight (**D**) cell density. Data are the mean of four independent replicates from production cultures after 24 h of induction with DNB medium, and the error bars represent the standard deviation. * BZS+Mev *E. coli* Top10 strain induced with 1 mM IPTG and different levels of arabinose (Ara).

For the overexpression of (+)-zizaene, the production cultures were performed by the two-phase partitioning method [31] with a DNB medium and an overlay of iso-octane, which reduces the loss of (+)-zizaene due to its volatility. After 24 h of culture, organic phases from production cultures were obtained and analyzed through GC-MS for the identification of terpenes. All tested strains produced (+)-zizaene as the main product (90%) and β-acoradiene as a side product (10%) (Figure 2), except for the Mev strain (negative control) because it lacks the *ZS* gene and harbors the MEV pathway (Figure 3a). Additionally, the Mev strain produced farnesol, which is a product from the hydroxylation of FDP by endogenous phosphatases from *E. coli* [32].

**Figure 4:**
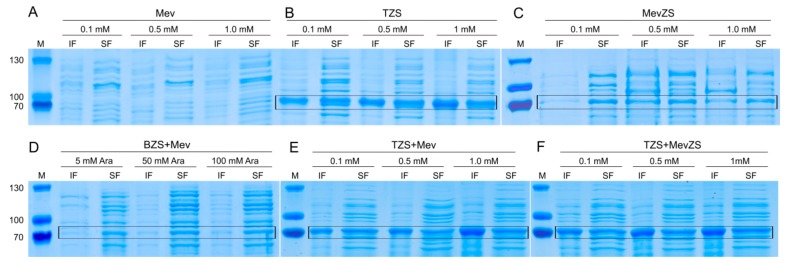
Evaluation of the overexpression of the soluble (SF) and insoluble (IF) ZS protein fractions from the engineered *E. coli* BL21(DE3) strains, induced at different IPTG levels by 10% SDS-PAGE. (M) molecular marker. * BZS+Mev induced with 1 mM IPTG and different levels of arabinose (Ara).

As a first attempt to produce (+)-zizaene, the TZS strain was constructed with the *ZS* gene under the control of the T7 promoter and the FDP supply originated from the endogenous MEP pathway from *E. coli* BL21(DE3) strain. As shown in Figure 3a, the (+)-zizaene titer of the TZS strain was the lowest when compared to all the tested strains; however, it achieved the highest cell growth (biomass 2.9 g L^−1^ and cell density of OD_600_ 7.2, Figure 3c,d) and amounts of soluble ZS protein (Figure 4b) for the TZS strain induced at 0.1 mM IPTG. These observations suggest a low metabolic burden due to the overexpression of a single recombinant protein (ZS). Moreover, it evidences the limited supply of the endogenous FDP from *E. coli*, which is required for the biosynthesis of isoprenoid quinones and peptidoglycans [13,14].

### 3.2. Improving the Titers of (+)-zizaene through the Mevalonate Pathway

To raise the supply of the precursor FDP, the polycistronic pMevZS vector was constructed with the *ZS* gene and the exogenous MEV pathway (Figure S1), which was obtained from the pMEV vector that harbors the following genes: *AtoB*, HMGS*, tHMGR*, MK*, PMK*, *PMD*, *IDI*, and *IspA* (*genes from *S. cerevisiae* that were codon-optimized to *E. coli*) [18]. Consequently, a single mRNA transcript was expressed under the control of the *lac*UV5 promoter. As a result, overexpression of the heterologous MEV pathway with the *ZS* gene (MevZS strain) induced with 0.1 mM IPTG augmented the (+)-zizaene production and yield by 2.7 and 5-fold respectively when compared to the TZS strain (Figure 3a,b). Thus, a relation was observed in both TZS and MevZS strains, where higher amounts of IPTG increased the overexpression of insoluble ZS protein (Figure 4a,c). Moreover, overexpression of the MEV pathway generated a strong metabolic burden, where cell growth diminished by 1.8-fold biomass and 2.1-fold cell density when compared to that of the strain lacking the MEV pathway (TZS strain) (Figure 3c,d). Although the overexpression of the MEV pathway raised the FDP supply, the production of soluble ZS protein declined dramatically when compared to the TZS strain (Figure 4c), suggesting that the ZS supply became the limiting factor.

### 3.3. Effect of Promoters on the Overexpression of the ZS Gene

The low ZS supply demonstrated by the MevZS strain could result from either expression mechanisms of polycistronic vectors and, or by the promoter strength. To prove these hypotheses, multi-vector strains were constructed harboring the following plasmids: polycistronic pMev vector (MEV pathway) and monocistronic ZS vector ((+)-zizaene pathway). Two versions of the ZS vectors were constructed in order to test the effect of promoters on the ZS supply. The designed ZS vectors comprised the pETZS vector with the strong T7 promoter and the pJbeiZS vector harboring the weak P_BAD_ promoter (Figure S2). The overexpression of the *ZS* gene under the control of the P_BAD_ promoter (BZS+Mev strain) did not enhance the (+)-zizaene titers when compared to the MevZS strain, but it did increase the (+)-zizaene yield by 2.5-fold when induced with 50 mM arabinose (Figure 3b). Nevertheless, the BZS+Mev strain showed the lowest cell growth of all the tested strains (biomass 0.63 g L^−1^ and cell density of OD_600_ 1.0, Figure 3c,d). Furthermore, overexpression of the *ZS* gene controlled by the T7 promoter (TZS+Mev strain) raised the amounts of soluble ZS protein when compared to that of the induced with the P_BAD_ promoter (Figure 4d,e). As a consequence, the (+)-zizaene production and yield augmented 7.2 and 4.4-fold, respectively when compared to the BZS+Mev strain, and 7.2 and 11.3-fold correspondingly when compared to the MevZS strain (Figure 3a,b). Such strategy improved the ZS supply and consequently restored the flux balance of the (+)-zizaene biosynthesis.

### 3.4. Enhancing the ZS Supply by Engineering Multiple Copies of the ZS Gene

Overexpression of the *ZS* gene with the T7 promoter seemed to overcome the ZS supply limitations of the MevZS and BZS+Mev strains. Nevertheless, the optimal amount of ZS to catalyze the FDP supply still remains unknown. To increase the ZS supply even further, a multi-vector strain that harbored two copies of the *ZS* gene was engineered. For that, the TZS+MevZS strain was developed, harboring the pETZS and pMevZS vectors, and controlling the expression of the *ZS* genes by the T7 and *lac*UV5 promoters. As a result, the multi-copy *ZS* gene strain achieved the best results of all the constructed strains (titer: 13.7 mg L^−1^ and yield 11.6 mg g^−1^ DCW) and raised 1.3-fold the (+)-zizaene titers and 1.6-fold the yields in comparison to the TZS+Mev strain (one *ZS* gene copy) (Figure 3a,b). Moreover, it augmented the (+)-zizaene production and titer by 9.7 and 18.4-fold correspondingly when compared to the single-vector MevZS strain. These results suggest that the bottleneck of the metabolic flux was still the ZS supply, which could be improved by the combination of the T7 promoter with multiple copies of the *ZS* gene, harbored on a multi-vector strain.

### 3.5. Optimization of Fermentation Conditions and Evaluation of E. coli Strains

**Figure 5:**
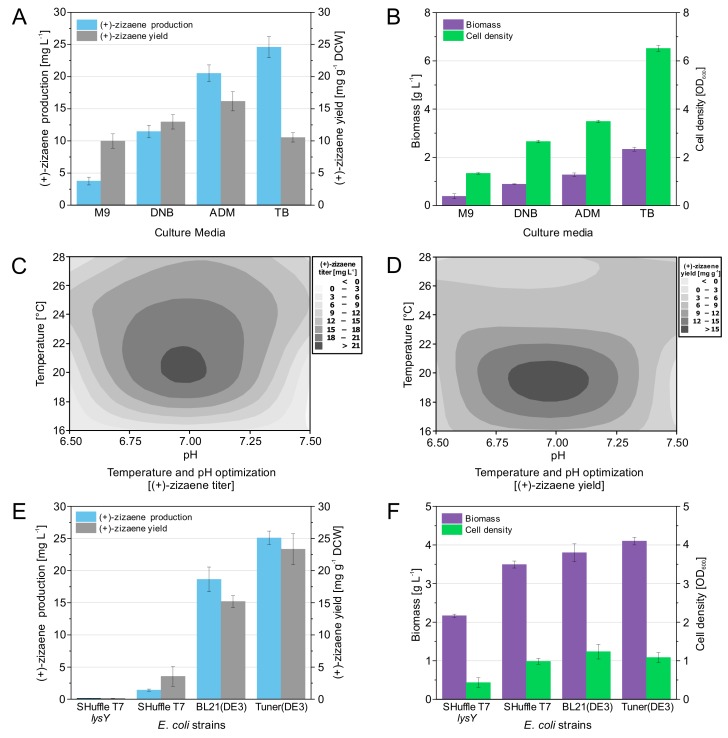
Optimization of the fermentation conditions and testing of *E. coli* strains for the improvement of the terpene performance. Evaluation of production media: (**A**) (+)-zizaene titers and yields, (**B**) biomass and cell density. Optimization of growth temperature and media pH: (**C**) (+)-zizaene titers, (**D**) (+)-zizaene yields. Evaluation of *E. coli* strains: (**E**) (+)-zizaene titers and yields, (**F**) biomass and cell density. Data are the mean of four independent replicates from production cultures after 24 h of induction, and the error bars represent the standard deviation.

To raise further the production of (+)-zizaene, fermentation tests were carried out at shake flask scale, comprising the evaluation of culture media, optimization of growth temperature and media pH, and testing of *E. coli* strains. Accordingly, the mineral-salt defined media DNB, ADM, and M9 were tested due to their previous use in metabolic engineering studies and suitability to control carbon-limited growth required for fed-batch or continuous cultivations to reach high-cell density cultures [33]. To compare the performance of defined media against complex media, the TB broth was used. As a result, substantial differences were observed between the defined media, where the highest (+)-zizaene levels and yields were obtained by ADM media, followed by DNB and M9 media (Figure 5a). Consequently, the ADM medium was 1.8-fold higher than the DNB medium for the (+)-zizaene titers and 1.2-fold higher for the (+)-zizaene yields. Both tested media were of similar formulation with the exception of the trace elements and nitrogen composition, whereas the concentration of nitrogen of the ADM medium was 2.5-fold higher than the DNB medium. Likewise, cell growth followed a similar behavior to that of the (+)-zizaene production among the tested media, where the complex TB broth had the highest cell growth when compared to the defined media tests (Figure 5b). However, the ADM medium obtained the highest (+)-zizaene yields (16.2 mg g DCW^−1^) when compared to all the tests and was consequently selected for further tests.

**Figure 6:**
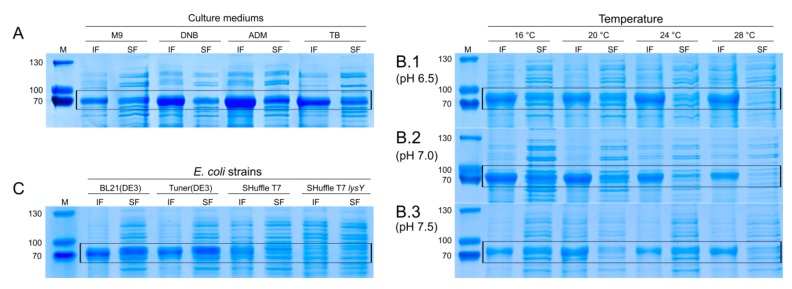
Analysis of the over-expression of soluble (SF) and insoluble (IF) ZS protein fractions from the optimization of fermentation conditions and testing of *E. coli* strains by 10% SDS-PAGE. (**A**) Culture media test. (**B**) Temperature and pH optimization. (**C**) *E. coli* strains test. (M) molecular marker.

To further improve the fermentation conditions, factorial designs of two levels were implemented for the optimization of growth temperature and pH of the ADM medium. Accordingly, optimal (+)-zizaene levels were found between 19.5‒21.8 °C and pH 6.8‒7.08 (Figure 5c) and for (+)-zizaene yields between 18‒20.8 °C and pH 6.8‒7.1 (Figure 5d). Temperature affected (+)-zizaene levels differently than yields, whereas moderate (+)-zizaene levels (15‒21 mg L^−1^) were shown at a broad range of temperature (17.5‒27.7 °C). On the contrary, pH had nearly the same effect on both variables. Additionally, the highest levels of soluble ZS protein were found at lower pH and temperatures (Figure 6b). Tests at pH 6.5 also produced the highest amounts of insoluble ZS protein, resulting in the lowest (+)-zizaene levels. Similarly, tests carried out at 16 °C dropped the (+)-zizaene levels despite the high amounts of soluble ZS protein, possibly due to enzymatic mechanisms. As a result, the optimal fermentation conditions were determined as 20 °C and pH 7.0, which improved the (+)-zizaene levels to 21.5 mg L^−1^ and yields to 16.2 mg g^−1^ DCW. 

Strain tests were carried out under optimal fermentation conditions (ADM medium, 20° C and pH 7) to assess the features of the *E. coli* B strains SHuffle T7, SHuffle T7 *lysY*, Tuner(DE3) and BL21(DE3), and measure their impact on the production of (+)-zizaene. The SHuffle-based strains were selected to test if there could be an enhancement in the folding of ZS due to the effect of a disulfide bond chaperone DsbC. Additionally, the SHuffle T7 *lysY* variant reduces the basal expression for toxic genes through the expression of lysozyme, coded by the *lysY* gene. Similarly, the BL21(DE3) variant, the Tuner(DE3) strain lacks the *lacY* gene that codes for the β-galactosidase permease, which allows fine-tuning for induction and reduces basal expression. As a control, the BL21(DE3) strain was used. As presented in Figure 5e, the differences between the BL21-based and SHuffle-based strains were remarkable; unexpectedly, the best results were shown by the Tuner(DE3) strain, followed by the BL21(DE3), SHuffle T7, and SHuffle T7 *lysY* strains. These observations demonstrate that the strains expressing the lowest number of recombinant proteins produced the highest cell growth (Figure 5f), and soluble ZS protein amounts (Figure 6c), and consequently, the highest (+)-zizaene titers and yields (shown by increasing number of recombinant proteins: Tuner(DE3): *ZS*; BL21(DE3): *ZS*, *lacY*; SHuffle T7: *ZS*, *lacY*, *DsbC*; SHuffle T7 *lysY*: *ZS*, *lacY*, *DsbC*, *lysY*). Overexpression of the chaperones by the SHuffle-based strains did not have the expected results; instead, it showed deleterious effects as observed from the low amounts of ZS protein. As a result, the strain that expressed the minimum number of recombinant proteins, the Tuner(DE3) strain, enhanced 1.2-fold the (+)-zizaene levels and 1.4-fold the yields in comparison with the control BL21(DE3) strain. 

Finally, the applied approach for the optimization of the fermentation conditions improved the (+)-zizaene titers 2.2-fold and yields 1.7-fold when compared to the strains without optimization (Section 3.4), achieving the highest (+)-zizaene production of 25.09 mg L^−1^ and yield 23.33 mg g^−1^ DCW for this study.

## 4. Discussion

The production of (+)-zizaene was metabolically engineered in *E. coli* by a recombinant codon-optimized SUMO-fused ZS that enhanced the amounts of soluble ZS protein [10]. Moreover, the optimization of the metabolic flux to (+)-zizaene was the greatest challenge, where the FDP and ZS supply were identified as the limiting factors. Consequently, a strategy was applied to gradually increase the supply of both and augment the metabolic rate driving force towards (+)-zizaene.

Initially, production of (+)-zizaene was possible by using the endogenous MEP pathway from *E. coli* (TZS strain) but at a lower extent. The high amounts of soluble ZS protein and low titers of (+)-zizaene suggest a limitation on the FDP supply. For that reason, the exogenous MEV pathway was applied, which is the ubiquitous pathway in plants for the biosynthesis of the sesquiterpene substrate FDP [34]. Hence, the pMevZS vector was constructed as a polycistronic vector, including all the required genes for terpene production into one vector. Such a strategy has been used for the engineering of many terpenes [23,28,35] because the maintenance of a single vector by the host cells is more efficient than for multi-vectors [36]. As a result, the overexpression of the MevZS strain did not increase the levels of (+)-zizaene as expected; possibly due to the lower amounts of soluble ZS protein than the TZS strain. This could be explained by the moderate strength of the *lac*UV5 promoter [37], theimproved variant of the *lac* promoter from *E. coli* [38]. Additionally, the *ZS* gene was placed eight genes downstream from the promoter in the polycistronic pMevZS vector and eventually, this gene order could affect the overexpression of ZS, as demonstrated by other studies [39,40,41]. Moreover, the overexpression of the nine genes produced a large mRNA transcript, which is usually more unstable as short transcripts [42]. Thus, the increase in the FDP supply and the decline in the soluble ZS protein amounts suggest that the limiting factor shifted to the ZS supply. This scenario was deleterious for an optimal metabolic rate, because ZS suffers substrate inhibition from FDP at higher levels of 5 µM, as shown in our previous report [20].

To raise the ZS supply, the monocistronic vectors pJbeiZS and pETZS were designed by cloning the *ZS* gene directly after the promoter and further co-transformed into the strain with the pMev vector. The use of the BZS+Mev strain was initially promising because it is capable of tuning the metabolic flux, using unique inducers for FDP (IPTG) and ZS (Arabinose). However, results with the P_BAD_ promoter were similar to those obtained by the MevZS strain, possibly due to the similarity in the strength of the P_BAD_ promoter versus the *lac*UV5 promoter [37]. Similar outcomes were demonstrated by others, where the *lac*UV5 and P_BAD_ promoters produced low or even undetectable levels of sesquiterpenes [11,12]. Additionally, accumulation of toxic intermediates due to a higher metabolic burden could occur because the BZS+Mev strain harbors the high-copy pJbeiZS plasmid with the pBBR1 origin of replication [43]. On the other hand, overexpression of soluble ZS protein with the T7-promoter (TZS+Mev strain) was drastically higher than with the P_BAD_ promoter (BZS+Mev strain) and subsequently, the production of (+)-zizaene increased considerably. 

We ventured to raise further the ZS supply by adding a second copy of the *ZS* gene through the TZS+MevZS strain. As a result, the production of (+)-zizaene was the greatest of all the engineered strains and suggests that the limiting factor was still the ZS supply. The improvement in (+)-zizaene levels could be due to the effect of overexpressing the *ZS* genes by two different promoters on two different vectors (T7 in pETZS and *lac*UV5 in pMevZS). Such strategy could have been a contributive factor, possibly by overexpressing the *ZS* gene by different promoters, by RBS strength differences from the used vectors, and by the vector copy numbers, which are unequal on multi-plasmid strains due to differences between replication origins, antibiotic markers, and plasmid sizes [16,42]. However, more tests are required to draw such conclusions. Additionally, it seems that the in vivo production of (+)-zizaene requires higher amounts of the TPS when compared to other sesquiterpenes such as vetispiradiene (K_M_ = 0.7 µM for the vetispiradiene synthase) [44], due to the low enzyme activity of ZS (K_M_ = 0.88 µM) [20]. Therefore, increasing the copy number of the TPS gene in a multi-vector strain was a plausible strategy to improve the flux to (+)-zizaene.

Optimal fermentation conditions are crucial for efficient microbial growth and metabolization of glucose flux towards terpenes, as demonstrated by many reports [11,45,46]. Regarding the media evaluation, the ADM medium achieved the highest production of (+)-zizaene when compared to the other defined media tests. The main difference in media formulation between tests was the higher amount of nitrogen from the ADM medium, suggesting that nitrogen concentration was the most relevant factor; particularly, because nitrogen is required for the biosynthesis of amino acids that regulates the production of cellular and recombinant proteins [47]. This could explain the higher cell growth and soluble ZS protein levels from the ADM medium tests when compared to the other tests, which in turn improved the (+)-zizaene levels. 

Optimization of the temperature and media pH can enhance the production of sesquiterpene as observed with the microbial production of vetispiradiene, 5-*epi*-aristolechene and (+)-δ-cadinene [11]. For the production of (+)-zizaene, the optimal fermentation conditions were found at 20 °C and pH 7.0, which differ from the optimal reaction conditions from the ZS in in vitro biotransformations with FDP (36 °C, pH 7.5) [20]. Thus, the greatest difference between the experimental conditions was the temperature, due to its relevant effect over protein synthesis rate, cell growth, and enzyme activity [11,48]. Accordingly, tests cultivated at 16 °C and pH 6.5‒7, overexpressed high amounts of soluble ZS protein but the (+)-zizaene levels and cell growth were low. These results suggest that the activity of the ZS was drastically reduced at 16 °C, due to its preference for higher temperatures (optimal at 36 °C). On the other hand, temperatures higher than 20 °C resulted in higher amounts of insoluble ZS protein than the soluble form. These observations confirm that an optimal temperature of 20 °C was required to achieve a balance between cell growth and active ZS protein. Regarding the effect of the media pH on (+)-zizaene production, the optimal pH was found to be neutral pH and values different than that lowered the (+)-zizaene titers. Additionally, lower pH values promoted the formation of insoluble ZS protein. Such behavior was observed before in uncontrolled-pH *E. coli* cultures grown in shake flasks, whereas overexpression of fused SpA-βgal protein at pH < 5.5 augmented the inclusion body formation, possibly due to regulatory mechanisms for pH homeostasis [49]. Therefore, tests grown at pH 7 at 20 °C achieved the balance between insoluble and soluble ZS protein fractions that led to the highest production of (+)-zizaene.

The evaluation of *E. coli* B strains revealed considerable differences in terpene production due to the particular feature of each strain. The terpene performance increased with the strains that expressed the lower number of recombinant proteins such as the BL21(DE3)-based strains. These results suggest that the expression of the ZS protein is not affected by basal expression (*lysY* gene), neither the β-galactoside permease is required (*lacY* gene), nor the chaperon DsbC enhanced the protein folding and disulfide bond formation, although the ZS protein contains cysteines. On the contrary, overexpression of DsbC drastically dropped the amounts of soluble ZS protein and the (+)-zizaene titers. Consequently, the Tuner(DE3) strain achieved the best performance for terpene production by overexpressing the minimum number of recombinant proteins, thus avoiding the production of unnecessary proteins (β-galactoside permease, DsbC, and lysozyme). This strategy diminished the metabolic burden, defined as the resources consumed for cell maintenance and recombinant protein synthesis that is inversely proportional to cell growth [48]. This was confirmed by the higher biomass amount from the Tuner(DE3) strain when compared to the other tested strains. As a result, the lower metabolic burden from the Tuner(DE3) strain improved the efficiency of the carbon flux towards (+)-zizaene.

## 5. Conclusions

In this work, we demonstrated the metabolic engineering of *E. coli* strains for the in vivo production of (+)-zizaene. Moreover, we optimized the limiting factors of the metabolic flux to (+)-zizaene by the overexpression of the MEV pathway, and by boosting the ZS supply by increasing the number of copies of the *ZS* gene with T7 promoters on a multi-plasmid strain. Final improvement was achieved by optimizing the fermentation conditions and testing of *E. coli* B strains to a final (+)-zizaene production of 25.09 mg L^−1^ at the shake flask scale. This work offers new insights into the optimization of the metabolic pathway and fermentation conditions for the in vivo production of sesquiterpenes and the development of a novel microbial platform towards the industrialization of fragrant molecules.

## Figures and Tables

**Table 1:** Strains and plasmids used in this investigation.

Plasmid Reference	Plasmid Name	Description (Origin of Replication, Promoter, Antibiotic Resistance and Genes)	Reference
pETZS	pETSUMO::ZIZ(co)	pBR322, PT7, Kan, harboring the codon-optimized SUMO-fused *ZS* gene from *Ch. zizanioides*	[10]
pMev	pBbA5c-MevT(CO)-MBIS (CO, ispA)	p15A, P*lac*UV5, Cam, harboring the mevalonate pathway genes: *AtoB*, *HMGS*, *tHMGR*, *MK*, *PMK*, *PMD*, *Idi* and IspA*.	[18]
pJbei-6411	pJbei-6411	pBBR1, P_BAD_, Kan, harboring the arabinose operon with the cytochrome P450 (CYP153A6) from *Sphingomonas sp*.	[23]
pMevZS	pBbA5c-MevT(CO)-MBIS (CO, ispA)-SUMO::ZIZ(co)	p15A, P*lac*UV5, Cam, harboring the mevalonate pathway genes* and the codon-optimized SUMO-fused *ZS* gene from *Ch. zizanioides*	This study
pJbeiZS	pJbei-6411-SUMO::ZIZ(co)	pBBR1, P_BAD_, Kan, harboring the arabinose operon and the codon-optimized SUMO-fused *ZS* gene from *Ch. zizanioides*	This study
**Strains**	**Genotype/Description**		**Reference**
*E. coli* TOP10	F^-^ *mcr*A *(mrr-hsd*RMS*-mcr*BC*)* ϕ*80lacZ ∆*M15 *∆lac*X74 *rec*A1 *ara∆*139 *(ara-leu)*7697 *gal*U *gal*K *rps*L (Str^R^) *end*A1 *nup*G		IBA
*E. coli* Bl21(DE3)	F^-^ *ompT hsdS_B_ (r*_B_*^-^m*_B_*^-^) gal dcm* (DE3)		Novagen
*E. coli* Tuner(DE3)	F^-^ *ompT hsdS_B_ (r*_B_*^-^m*_B_*^-^) gal dcm lacY1*(DE3)		Novagen
*E. coli* NEB 10-beta	Δ*(ara-leu) 7697 araD139 fhuA* Δ*lacX74 galK16 galE15 e14-* ϕ*80*d*lacZ*Δ*M15 recA1 relA1 endA1 nupG rpsL* (StrR) *rph spoT1* Δ*(mrr-hsdRMS-mcrBC)*		NEB
*E. coli* SHuffle T7	*fhuA2 lacZ::T7 gene1* (lon) *ompT ahpC gal λatt:*:pNEB3-r1-*cDsbC* (Spec^R^, *lacI^q^*) Δ*trxB sulA11 R(mcr-73::miniTn10*--Tet^S^)2 (dcm) *R(zgb-210::Tn10* --Tet^S^) *endA1 ∆gor ∆(mcrC-mrr)114::IS10*		NEB
*E. coli* SHuffle T7 *lysY*	MiniF *lysY* (Cam^R^)/*fhuA2 lacZ::T7 gene1* (lon) *ompT ahpC gal* *λatt:*:pNEB3-r1-*cDsbC* (Spec^R^, *lacI^q^*) Δ*trxB sulA11 R(mcr-73::miniTn10*--Tet^S^)2 (dcm) *R(zgb-210::Tn10* --Tet^S^) *endA1 ∆gor ∆(mcrC-mrr)114::IS10*		NEB
Mev	*E. coli* Bl21(DE3) harboring pMev		This study
ZS	*E. coli* Bl21(DE3) harboring pETZS		This study
MevZS	*E. coli* Bl21(DE3) harboring pMevZS		This study
BZS+Mev	*E. coli* TOP10 harboring pMev and pJbeiZS		This study
TZS+Mev	*E. coli* Bl21(DE3) harboring pMev and pETZS		This study
TZS+MevZS	*E. coli* Bl21(DE3) harboring pMevZS and pETZS		This study
T-TZS+MevZS	*E. coli* Tuner(DE3) harboring pMevZS and pETZS		This study
SH-TZS+MevZS	*E. coli* SHuffle T7 harboring pMevZS and pETZS		This study
SHL-TZS+MevZS	*E. coli* SHuffle T7 *lysY* harboring pMevZS and pETZS		This study

Kan, kanamycin and Cam, chloramphenicol.

**Table 2:** Primers used in this investigation.

Primers	Description	Reference
ZS-F-Mev	catccagcgtaataaataagGATCTAGGAGGTAATGGGCAGCAGCCATCATC	This study
ZS-R-Mev	gagatccttactcgagtttgTCACACCGGAATCAGATTTACATAC	This study
pJ6411-F-ZS	cccaagattacgtacattg	This study
pJ6411-R-ZS	ttctttatcctcctagatcttttgaattcccaaaaaaacg	This study
ZS-F-pJ6411	ccgtttttttgggaattcaaaagatctaggaggataaagaaATGGGCAGCAGCCATCATC	This study
ZS-R-pJ6411	tcaatgtacgtaatcttgggTCACACCGGAATCAGATTTACATAC	This study

Upper case sequences: Inserts. Lower case sequences: backbone vector. Upper case underlined sequences: IRES

## Supplementary Materials

The following are available online at https://www.mdpi.com/2073-4425/10/6/478/s1, Figure S1: Confirmation of seamless cloning for pMevZS; Figure S2: Confirmation of seamless cloning for pJbeiZS; Table S1: Mass spectra of the identified products.
