# Supplementary material for: Modulating the Precursor and Terpene Synthase Supply for the Whole-Cell Biocatalytic Production of the Sesquiterpene (+)-Zizaene in a Pathway Engineered E. coli
Source: Genes (Basel). 2019 Jun 24;10(6):478. doi: 10.3390/genes10060478 (PMC6627501; doi:10.3390/genes10060478)
Supplement: Supplementary file 1 [file genes-10-00478-s001.pdf]

Supplementary Materials

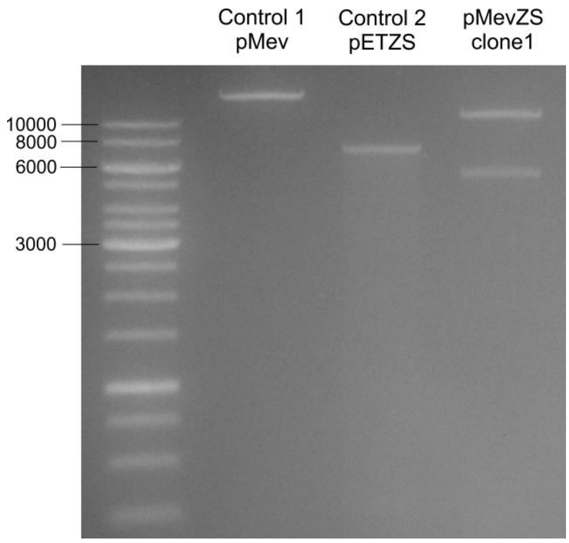

Figure S1. Confirmation of the cloning of the pMevZS by restriction digestion with BglII. Controls shows only one fragment with the respective backbone sizes: pMev 13.1 kb and pETZS 7.3 kb. The vector pMevZS contains two fragments of 5.3 kb and 9.8 kb resulting in a final size of 15.1 kb.

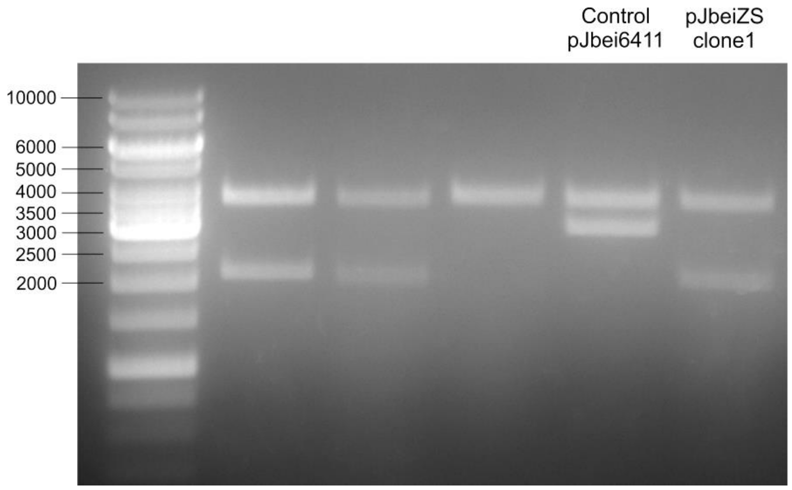

Figure S2. Confirmation of the seamless cloning of the pJbeiZS by double restriction digestion with BglII and XhoI. Vector pJbeiZS shows the 4 kb pJbei6411backbone and the 2 kb insert *ZS* gene. Similar digestion of control vector pJbei6411 appears with similar backbone size and with an insert cassette of 3 kb consisting of the cytochrome P450 operon (*ahpG*, *ahpH* and *ahpI* genes).

Table S1. Comparison of mass spectra and retention indices (RI) between the *in vivo* production of sesquiterpenes ((+)-zizaene and β-acoradiene) and references: authentic sesquiterpenes from the vetiver oil and database references.

(+)-zizaene	β-acoradiene
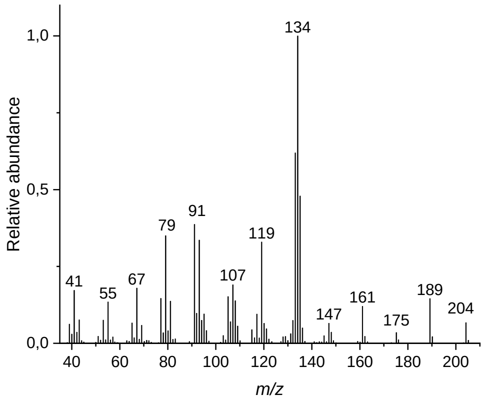 (+)-zizaene from *in vivo* production system<br>RI: 1617	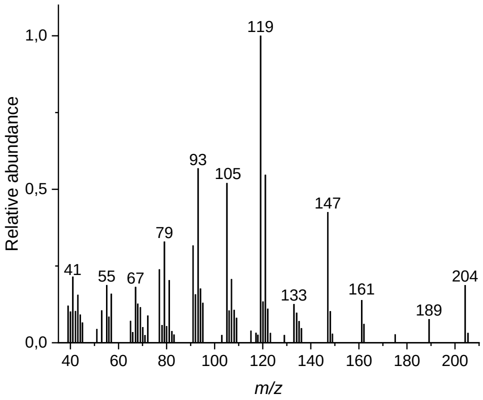 β-acoradiene from *in vivo* production system<br>RI: 1670
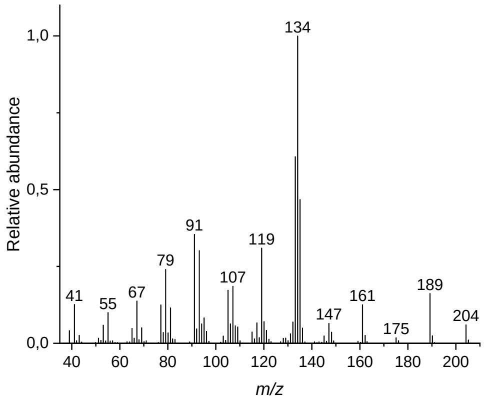 (+)-zizaene from authentic standard<br>from the vetiver oil. RI: 1617	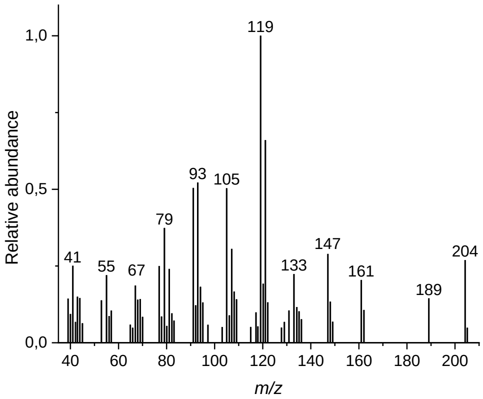 β-acoradiene from authentic standard<br>from the vetiver oil. RI: 1670
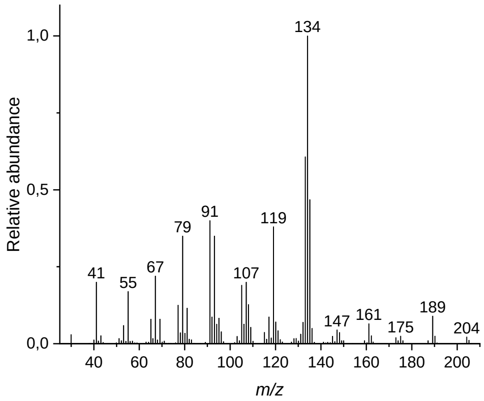 (+)-zizaene from database reference. RI: 1620<br>(Martinez et al., 2004)	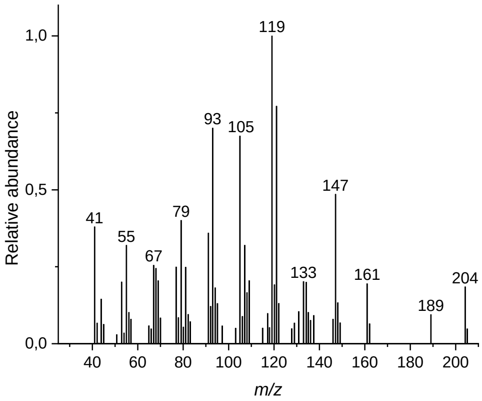 β-acoradiene from database reference. RI: 1672<br>(Martinez et al., 2004)
